# Efficacy and safety of infliximab and adalimumab in inflammatory bowel disease patients

**DOI:** 10.1007/s10787-024-01508-w

**Published:** 2024-07-10

**Authors:** Mahmoud E. Kamal, Rehab H. Werida, Mahasen A. Radwan, Safaa R. Askar, Gamal A. Omran, Marwa A. El-Mohamdy, Radwa S. Hagag

**Affiliations:** 1 Clinical Pharmacy and Pharmacy Practice Department, Faculty of Pharmacy, Egyptian Russian University, Cairo, Egypt; 2 Clinical Pharmacy and Pharmacy Practice Department, Faculty of Pharmacy, Damanhour University, Damanhour, Egypt; 3 Tropical Medicine Department, Faculty of Medicine, Ain Shams University, Cairo, Egypt; 4 Biochemistry Department, Faculty of Pharmacy, Damanhour University, Damanhour, Egypt; 5 Clinical Pathology Department, Faculty of Medicine, Ain Shams University, Cairo, Egypt

**Keywords:** Inflammatory bowel disease, Infliximab, Adalimumab, Efficacy, Safety, CDAI, DAI, sTREM-1

## Abstract

**Introduction:**

Inflammatory bowel disease (IBD), consists of two primary types: Ulcerative Colitis (UC) and Crohn’s Disease (CD). Infliximab (IFX) and Adalimumab (ADA) are frequently utilized in the management of moderate to severe cases of IBD.

**Aim:**

This study aimed to assess the efficacy and safety of IFX and ADA in individuals diagnosed with moderate to severe IBD.

**Method:**

This study is a prospective open-labeled randomized parallel study that included moderate to severe IBD patients treated with either IFX or ADA. A total of 56 patients participated, with 34 patients received IFX and 22 patients received ADA. Various measures, including Crohn’s Disease Activity Index (CDAI), Mayo Score/ Disease Activity Index (DAI), and C-reactive protein (CRP) levels, were taken at baseline and week 14 to assess the efficacy of the treatments. In addition, the levels of drugs and sTREM-1 were measured at 14 weeks. Patient safety was monitored throughout the study period.

**Results:**

In the group received IFX, there was a notable decrease in CDAI (*P* = 0.045), DAI (*P* = 0.026), and CRP (*P* = 0.023 for CD, and *P* = 0.021 for UC) levels. In addition, the group received ADA experienced a significant reduction in CDAI (*P* = 0.001), DAI (*P* = 0.032), and CRP (*P* < 0.018 for CD and *P* = 0.003 for UC) levels. Responders had higher drug concentrations than non-responders, notably IFX concentration was higher in responders with CD (*P* = 0.001) and UC (*P* < 0.001). ADA concentration was higher in UC (*P* <= 0.001) and all CD patients responded to the treatment. The same trend was observed for sTREM-1 levels in CD and UC patients (*P* = 0.042, and *P* = 0.015, respectively) in the IFX group. In UC patients treated with ADA, the level of sTREM-1 was significantly low (*P* = 0.002).

**Conclusion:**

Both IFX and ADA have a good safety profile and deliver a beneficial clinical and laboratory response in moderate-severe IBD patients.

**Clinical Trial Registration:**

This study is registered on ClinicalTrials.gov under the identifier NCT05291039. (You can access the study at https://clinicaltrials.gov/study/NCT05291039 (First Posted: March 22, 2022).

## Introduction

Inflammatory bowel disease (IBD) is a long-term inflammatory condition that affects the digestive system, which includes ulcerative colitis (UC) and Crohn’s disease (CD). It can affect people of all ages and genders (Aardoom et al. 2019). Symptoms of IBD include ulceration, inflammation of the tissues of the GIT, diarrhea, abdominal pain, anemia, weight loss, and rectal bleeding (Aardoom et al. 2019).

Diagnosis of IBD depends on inflammatory markers, clinical findings, and endoscopic evaluation. An inflammatory marker is C-reactive protein (CRP), which rises when there is inflammation in the intestine (Afif et al. 2009).

IBD complications are divided into two categories, extra-intestinal and intestinal complications. Extra-intestinal complications are osteoporosis, arthritis, anemia, aphthous ulcers, uveitis, anal fistulas, and erythema nodosum. Intestinal complications include colon cancer and colon perforation (Afif et al. 2009).

The severity of CD is determined using the Crohn’s Disease Activity Index (CDAI), which helps to evaluate the stage of CD. In contrast, UC is evaluated based on the Mayo score/disease activity index (DAI). CDAI ranges from 0 to 600. CDAI scores are based on symptoms, signs, blood test results, patient demographic characteristics, and extra-intestinal findings (Afify et al. 2021; Albader et al. 2021).

The DAI is a scale that goes from 0 to 12, and each part is ranked from 0 to 3. The DAI score is calculated by looking at symptoms like rectal bleeding, stool frequency, doctor’s evaluation, and endoscopy results (Albader et al. 2021; Cholapranee et al. 2017).

The treatment approach for IBD aims to relieve symptoms and achieve mucosal healing through a step-up approach of medication. Amino-salicylates are the first-line treatment, followed by corticosteroids if there is no response. If symptoms persist or recur during corticosteroid tapering, a purified protein derivative test is conducted to rule out latent tuberculosis before starting immune-modifying agents (Anti-TNF agents). If no response is achieved, surgery may be necessary (Colombel et al. 2009; Da et al. 2013).

Infliximab (IFX) and adalimumab (ADA) are examples of approved anti-TNF agents (biologic drugs) that are used in the management of IBD. Both IFX and ADA are effective and safe for managing IBD in the short and long term (Domenicantonio et al. 2018).

Soluble triggering receptor expressed on myeloid cells-1 (sTREM-1), a protein found on specific immune cells, is known to increase in inflammatory situations. Recent research has shown higher levels of sTREM-1 in individuals with IBD which is potentially tied to the severity of the disease (Doecke et al. 2017).

The Egyptian Ministry of Health introduced recently IFX and ADA in the treatment protocol for IBD. Although the prevalence of IBD is considered at a low rate in Egypt, the curve of newly diagnosed cases is increasing and there are a few data regarding the management of biologic treatments in Egyptian IBD patients (Gisbert and Chaparro 2021). Therefore, this research aims to assess the efficacy and safety of IFX and ADA in treating IBD Egyptian patients.

## Design and methods

Our study was conducted on patients with IBD who were split into two treatment groups, IFX and ADA, in a prospective, parallel, randomized manner. The study assessed trough concentrations of IFX and ADA, sTREM-1 level, CRP levels, CDAI, and DAI score. Trough concentrations of IFX and ADA were assessed after 14 weeks of starting treatments. Then Patients were further followed up for 2 months. Follow-ups were performed via scheduled hospital visits and phone calls for all enrolled patients.

### Study population and eligibility

All patients with IBD were selected from the Outpatient Clinic at Ain Shams University Hospitals in Cairo, Egypt, and were evaluated for eligibility. The criteria for inclusion and exclusion can be found in Table 1. Six hundred and seven patients presented to the clinic. Eligible patients were randomly assigned based on the appointment number.

**Table 1:** Inclusion and exclusion criteria

Inclusion criteria	Exclusion criteria
Adult patients age between 18 and 65 years Patients diagnosed with moderate to severe IBD according to ECCO guidelines (4) IBD patients receiving either IFX or ADA	Patients who missed follow-up for 1 month Non-compliant patients to the treatment regimen Patients having any contraindications to the biologic therapy e.g.: latent TB, a viral or fungal or bacterial infection

*IFX* Infliximab, *ADA* Adalimumab, *IBD* Inflammatory bowel disease, *TB* Tuberculosis and *ECCO* European Crohn’s and Colitis Organization

**Fig. 1:**
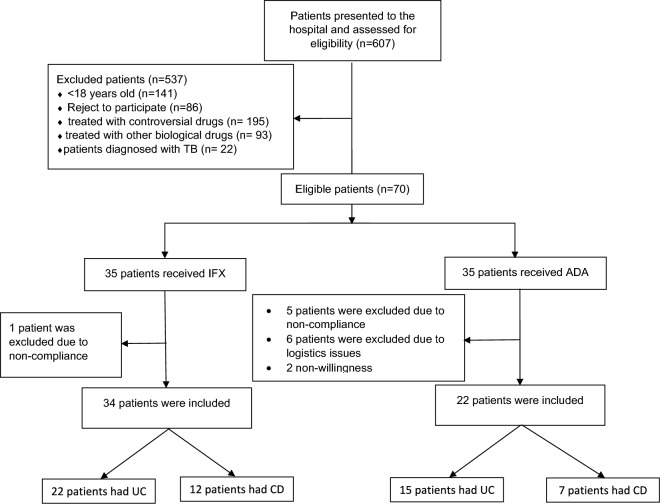
Flow diagram for the IBD patients included in the study. *UC* ulcerative colitis, *CD* Crohn’s disease, *TB* Tuberculosis

All eligible patients were asked to provide a written informed consent for participation in the study. Figure 1 is a flow diagram for the patients included in the study.

### Intervention

The loading dose of ADA (Humira^®^, AbbVie pharmaceuticals, North Chicago, USA) is 160 mg subcutaneously, given either as 4 injections of 40 mg on the first day or as 2 injections of 40 mg daily for 2 consecutive days, followed by a dose of 80 mg subcutaneously 2 weeks later. The maintenance dose is 40 mg subcutaneously every 2 weeks.

For IFX (Remicade^®^, Janssen Biotech pharmaceuticals, Horsham Township, Pennsylvania, USA), the loading dose is 5 mg/kg intravenously at 0, 2, and 6 weeks, with a maintenance dose of 5 mg/kg intravenously every 2 months.

In patients previously treated with biologic drugs there were washout periods of 4 weeks in the case of IFX and 5 weeks in the case of ADA or golimumab (Han et al. 2020).

### Compliance

During the trial, a clinical pharmacist monitored compliance by ensuring patients received their scheduled doses at each visit. In addition, two phone calls were made to follow up with the patients. A colonoscopy was performed at baseline and week 14, using Olympus^®^ CF-H180, Japan, to assess the patient’s condition in both treatment groups.

### Assessment plan

Baseline assessments included patient history and current symptoms, CRP levels, CDAI, DAI, and colonoscopy. At week 14, CRP, CDAI, DAI, and colonoscopy were reassessed for all patients. sTREM-1 and trough concentration levels of IFX and ADA were also assessed at week 14. Patients were further followed up for 2 months for any side effects.

### Primary endpoint

Assessment of efficacy was performed according to; trough concentrations of IFX and ADA, CRP, CDAI score, DAI score, and serum TREM-1 for all included patients.

### Determination of trough concentrations of the biologic drugs (infliximab and adalimumab) and level of soluble triggering receptor expressed on myeloid cells-1 in the serum

After 14 weeks of treatment with either IFX or ADA regimens, blood samples (5 ml) were collected before the next dose (trough concentration). Serum trough concentration of IFX and ADA were measured using ELISA kits (RIDASCREEN^®^ IFX Monitoring, R-Biopharm AG^®^, Darmstadt, Germany, and RIDASCREEN^®^ ADA Monitoring, R-Biopharm AG^®^, Darmstadt, Germany, respectively). The serum TREM-1 was measured using the Human Triggering Receptor Expressed on Myeloid Cells-1 ELISA Kit (Bioassay Technology Laboratory, Shanghai, China). ELISA kits were used according to the manufacturer’s rules.

### Determination of efficacy by crohn’s disease activity index

Patients diagnosed with moderate to severe active CD underwent treatment with either IFX or ADA and were assessed for effectiveness using the CDAI. Clinical response (CR) was evaluated based on CR-70 and CR-100 criteria, signifying a decrease of at least 70 points and 100 points, respectively in CDAI scores from the initial assessment (Han et al. 2020).

### Determination of efficacy by mayo score/disease activity index

The CR was determined by a decrease of at least 3 points and 30% from the initial Mayo score, as well as a decrease of at least 1 point in the rectal bleeding sub-score or reaching an absolute rectal bleeding sub-score of 0 or 1 (Kamat et al. 2019).

### Secondary endpoint

Any adverse effect was recorded during the follow-up visits or calls for the included patients in both regimens as summarized in Table 2.

**Table 2:** Side effects in IFX and ADA treatment groups

Side effect	IFXN (%) (*N* = 34)	ADAN (%) (*N* = 22)	*P* value
Fever	3 (8.8)	1 (4.5)	0.485
Muscle pain	6 (17.6)	2 (9.1)	0.315
Infusion reaction	4 (11.8)	2 (9.1)	0.560
Flu-like symptoms	2 (5.9)	1 (4.5)	0.661
Hair loss	1 (2.9)	1 (4.5)	0.636
Severe constipation	1 (2.9)	0	N/A
Arthralgia	1 (2.9)	0	N/A
GERD	1 (2.9)	0	N/A
Skin lesion	0	1 (4.5)	N/A
Fatigue	6 (17.6)	1 (4.5)	0.151
Headache	4 (11.8)	2 (9.1)	0.560

Data are compared by Chi-squared test

*N* number of patients, *IFX* infliximab, *ADA* adalimumab, and *GERD* Gastroesophageal reflux disease

The *P* value is statistically significant at < 0.05 (1-sided significant)

### Statistical analysis and sample size

Statistical analysis was conducted using the Statistical Package of Social Sciences (SPSS) software version 20. A normality test was carried out for continuous variables. If the data was normally distributed, paired samples *t* tests were used to compare the mean and standard deviation before and after treatments. Unpaired t tests were used for comparisons between groups. Otherwise, non-parametric data was compared using the Mann–Whitney *U* test and the Wilcoxon signed-rank test. For non-parametric data, patient demographics and baseline characteristics were described using descriptive statistics. Continuous variables were presented as means ± SD, while categorical variables were presented as frequencies and percentages. The Chi-square test was used to compare responses between the two treatment groups for categorical data. The level of significance was considered significant when *P* values < 0.05.

### Ethical considerations

The trial had no impact on the patient’s treatment or follow-up.

### Trial status

This study followed the ethical guidelines outlined in the Declaration of Helsinki and the obtained approval from the Faculty of Medicine at Ain Shams University Hospitals, and Damanhur University—Faculty of Pharmacy—Research Ethics Committee with an ethical approval number FMASU R175/2023 and 923PP68M, respectively. The study is registered on ClinicalTrials.gov with the identifier NCT05291039. Participants in the study provided informed consent to participate. (First Posted: March 22, 2022). Visit the study page at: https://clinicaltrials.gov/study/NCT05291039. Patients’ inclusion began in December 2021 and ended in March 2023.

## Results

Tables 3 and 4 provide an overview of the baseline clinical features and demographics of the studied patients. Meanwhile, Table 5 outlines the clinical characteristics of the treatment groups receiving IFX and ADA before and after therapy. In our study, four patients out of the 56 included in both groups IBD patients who needed hemicolectomy, two CD patients, and two UC patients. Similarly, two CD patients needed bowel resection. In addition, one patient out of the 12 CD patients underwent ileocecal mass removal surgery.

**Table 3:** Baseline characteristics of patients

	Adalimumab (*N* = 22)	Infliximab (*N* = 34)
	Number (%)	Number (%)
Disease		
UC	15 (68.2)	12 (35.3)
CD	7 (31.8)	22 (64.7)
Anal fistula	5 (22.7)	10 (29.4)
Fever	2 (9.1)	11 (32.4)
Erythema	8 (36.4)	13 (38.2)
Extra-intestinal	8 (36.4)	7 (20.6)
Arthritis	7 (31.8)	7 (20.6)
Uveitis	2 (9.1)	0 (0)
Arthralgia	9 (40.9)	16 (47.1)
Smoking	2 (9.1)	5 (14.7)

**Table 4:** Demographics of the included patients

	Adalimumab (*N* = 22)	Infliximab (*N* = 34)	*P* value
	Number (%)	Number (%)	
Age (mean ± SD)			
UC	34.35 ± 10.22	32 ± 7.84	0.258
CD	36 ± 12.32	29.16 ± 6.97	0.205
Sex			
Male	10 (45.5)	18 (52.9)	0.392
Female	12 (54.5)	16 (47.1)	0.392
Marital status			
Married	16 (72.7)	20 (58.8)	0.22
Single	6 (27.3)	14 (41.2)	0.22
Occupation			
professional	6 (27.3)	11 (32.4)	0.315
Manual	2 (9.1)	6 (17.6)	0.315
Student	2 (9.1)	1 (2.9)	0.339
Not working	12 (54.5)	16 (47.1)	0.392
Previous biologics			
IFX	4 (18.2)	0 (0)	n/a
ADA	0 (0)	1 (2.9)	n/a
GOM	0 (0)	1 (2.9)	n/a
Previous treatment			
CORT	1 (4.5)	2 (5.9)	0.661
5-ASA	1 (4.5)	1 (2.9)	0.636
CORT + 5-ASA + immunomodulator	14 (63.6)	26 (76.5)	0.230
CORT + 5-ASA	2 (9.1)	2 (5.9)	0.515
Immunomodulators + 5-ASA	1 (4.5)	1 (2.9)	0.636
GOM + CORT + 5-ASA + immunomodulator	0 (0)	1 (2.9)	n/a
ADA + CORT + 5-ASA + Immunomodulator	0 (0)	1 (2.9)	n/a
CORT + 5-ASA + IFX	4 (18.2)	0 (0)	n/a

Data are compared by Chi-squared test

*UC* ulcerative colitis, *CD* Crohn’s disease, *IFX* infliximab, *ADA* adalimumab, *GOM* Golimumab, *CORT* Corticosteroids, and *5-ASA* aminosalicylates

*P value is statistically significant at < 0.05

**Table 5:** Clinical characteristics of infliximab and adalimumab treatment groups before and after therapy

	Infliximab (*N* = 34)						Adalimumab (*N* = 22)					
	CD	^a^P	^b^P	UC	^a^P	^b^P	CD	^a^P	^b^P	UC	^a^P	^b^P
CRP level before (mg/L)	30.89 (20–107.8)	0.485	0.023	41.11 ± 35.22	0.602	0.021	15 (3.4–93.5)	N/A	0.018	37.96 ± 27.43	0.252	0.003
CRP level after treatment (mg/L)	20.55 ± 15.68	0.002		18 (3–77)	0.002		3.65 ± 2.4	N/A		16.6 ± 12.8	< 0.001	
CDAI before treatment	327.2 ± 93.3	0.211	0.045	N/A	N/A	N/A	346.4 ± 52.4	N/A	0.001	N/A	N/A	N/A
CDAI after treatment	298.5 ± 87.7	0.025		N/A	N/A		216.1 ± 52.6	N/A		N/A	N/A	
DAI before treatment	N/A	N/A	N/A	9 (5–12)	0.815	0.026	N/A	N/A	N/A	9.7 ± 1.98	0.129	0.032
DAI after treatment	N/A	N/A		7 (4–12)	< 0.001		N/A	N/A		7.8 ± 2.5	0.008	
Medication concentration (ug/ml)	3.45 ± 3.04		0.001	2.41 ± 1.49		< 0.001	6.51 ± 3.68		N/A	2.1 (0–18.7)		0.001
sTREM-1 after treatment (ng/L)	264.8 (116–811)		0.042	332.75 ± 240.502		0.015	264 (11.09–2400)		N/A	261.16 ± 133.23		0.002

Parametric data are presented as mean ± SD and non−parametric data are summarized as median (range). *P* value Statistically significant at < 0.05. N is the number of patients

*N* number of patients, UC: ulcerative colitis; *CD* Crohn’s disease, *CRP* C-reactive protein, *CDAI* Crohn’s disease activity index, *DAI* Mayo score/disease activity index and *sTREM-1* Serum triggering receptor expressed on myeloid cells

^a^P: *P* value Comparing the value of parameters between patients who showed efficacy versus patients who did not show efficacy. ^b^P: *P* value comparing data between baseline and week 14

In our study, two IBD patients were diagnosed with rheumatoid arthritis, one patient had myelophthisic anemia, one patient had Mediterranean fever with thalassemia, and one patient had hypothyroidism.

### Efficacy

#### Crohn’s disease activity index

**Fig. 2:**
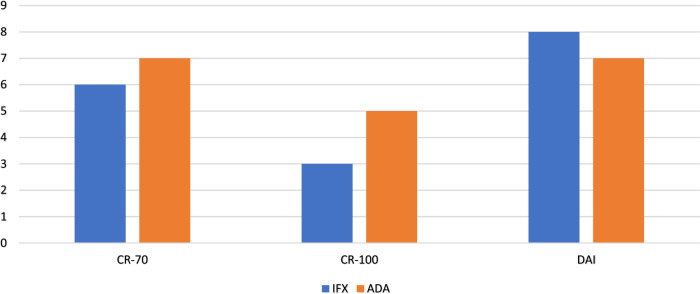
Number of CD and UC patients who showed a clinical response for IFX and ADA according to CR-70, CR-100, and DAI. *CR* clinical response, *DAI* Mayo Score/Disease Activity Index, *IFX* Infliximab, *ADA* Adalimumab

Figure 2 displays the number of patients who achieved CDAI response with IFX and ADA after 14 weeks in patients with CD. There was a significant decrease in scores for IFX (*P* = 0.045) and ADA (*P* = 0.001). ADA displayed effectiveness rates of 100 and 71.4% based on CR-70 and CR-100, respectively. In comparison, IFX exhibited effectiveness rates of 50 and 25% according to CR-70 and CR-100, respectively.

#### Mayo score/disease activity index

According to the Mayo score, ADA and IFX showed efficacy with 46.66 and 36.36%, respectively. Figure 2 illustrates the number of patients who achieved a clinical response to IFX and ADA after 14 weeks of treatment for UC. Both groups showed a significant decrease in their scores, with IFX having a *P* value of 0.026 and ADA having a *P* value of 0.032.

#### Medication Concentration

**Fig. 3:**
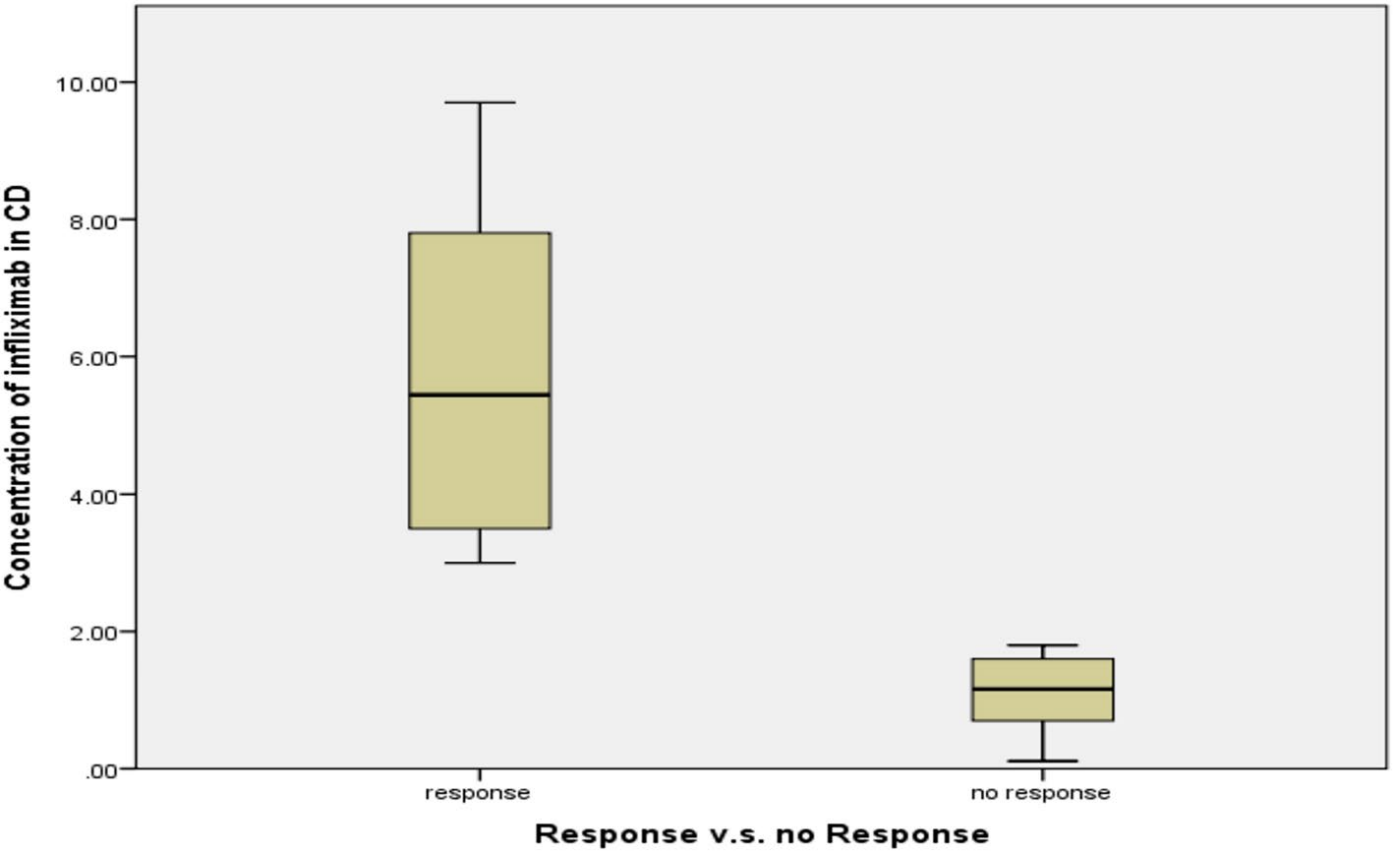
Levels of IFX in responsive and non-responsive CD patients

**Fig. 4:**
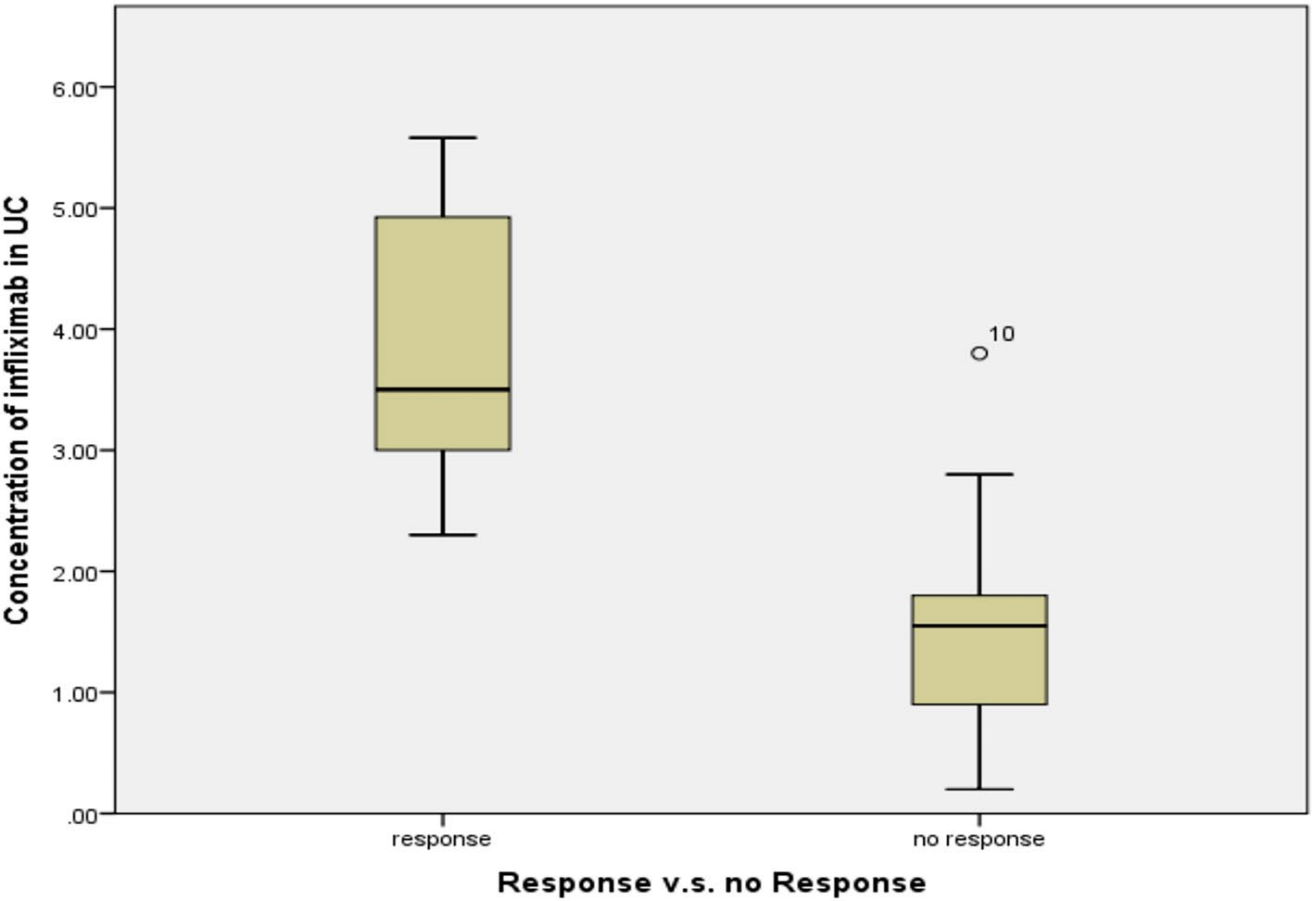
Levels of IFX in responsive and non-responsive UC patients

Figure 3 shows the levels of IFX in CD patients who responded and didn’t respond to treatment. Patients who achieved a clinical response based on CDAI-70 had significantly higher levels of IFX (5.8 ± 2.6 μg/mL) compared to non-responding patients (1.1 ± 0.7 μg/mL) group (*P* = 0.001). The same trend was observed with UC patients treated with IFX as seen in Fig. 4.

**Fig. 5:**
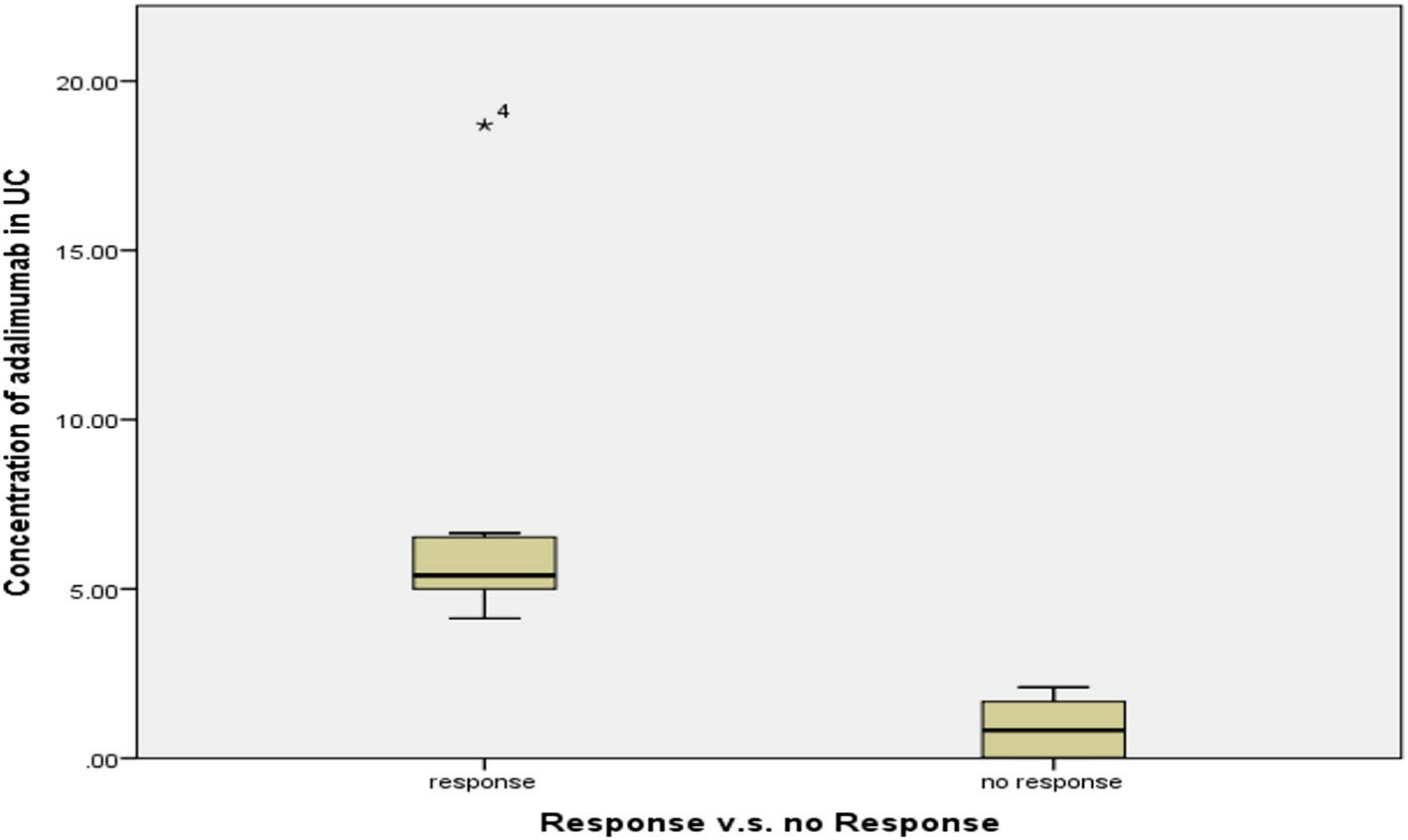
Levels of ADA in responsive and non-responsive UC patients

In Fig. 5, it is shown that patients with ulcerative colitis who experienced an improvement in their symptoms had significantly elevated serum levels of ADA (7.3 ± 5 μg/mL) in comparison to those who did not show a similar response. (0.9 ± 0.9 μg/mL) according to DAI (*P* < 0.001). One outlier was noticed in the responded group with a serum level of 18.7 μg/mL of ADA.

It was noticed that ADA’s mean level in CD patients was 6.5 ± 3.7 μg/mL with no “non-response” patients.

#### Levels of C-reactive protein

In Table 5, the levels of CRP before and after treatments for patients with IBD were compared.

After treatment, the CRP levels significantly decreased in both the IFX and ADA groups, with a *P* value less than 0.05.

#### Soluble triggering receptor expressed on myeloid cells-1 (sTREM-1)

Table 5 also shows the levels of sTREM-1 for patients with UC and CD. Patients who received IFX and showed clinical response had significantly lower levels of sTREM-1 compared to those who did not respond. ADA also had a similar impact on patients with CD and UC.

#### Adalimumab and infliximab

The clinical response to ADA and IFX was measured using CDAI and DAI for CD and UC patients, respectively. In CD patients, ADA showed a higher clinical response compared to IFX (*P* = 0.034). In UC patients, both ADA and IFX showed similar effectiveness (*P* = 0.386).

### Safety

The main side effects that were reported included headache, tiredness, muscle pain, and reactions from the infusion. Out of the 34 people who received IFX, 6 (17.6%) felt fatigued after the treatment, while only 1 out of 22 (4.5%) ADA recipients experienced fatigue. Infusion reactions, which showed symptoms like headache, dizziness, nausea, irritation at the injection site, flushing, chest pain, and difficulty in breathing, happened in 4 out of 34 people who received IFX (11.8%). Most of these reactions were not severe. Pre-treatment with steroids effectively managed this side effect. Since ADA was injected under the skin, reactions at the injection site included pain, sensitivity, redness, hardening, and swelling. This happened in 2 out of 22 (9.1%) patients. Muscle pain was observed in 17.6% of patients in the IFX group and in 9.1% of patients in the ADA group. The pain was felt in different parts of the body like the back, neck, and limbs. Headache was also noted in 11.8% of patients in the IFX group and in 9.1% of patients in the ADA group, occurring several hours after treatment.

The pregnant patient who received IFX did not experience any negative effects during her pregnancy. She responded well to IFX and stopped taking the medication 2 months before giving birth without any issues.

## Discussion

The Egyptian Ministry of Health recently approved the use of IFX and ADA for treating moderate to severe IBD. This research project aims to evaluate the effectiveness and safety of both treatments in Egyptian IBD patients.

Our findings show that IBD patients treated with IFX or ADA experienced a significant reduction in sTREM-1, CRP levels, CDAI, and DAI. Patients who responded well to the treatment had serum therapeutic levels of IFX and ADA, while those who did not respond adequately had sub-therapeutic levels. Furthermore, our research indicated that the effectiveness of IFX and ADA in patients with CD and UC is dependent on the concentration of the drugs in their body. This discovery aligns with previous studies conducted on a larger scale (Kamat et al. 2019; Kawalec et al. 2013; Kim et al. 2020).

It was noted that only one of the patients treated with ADA (4.5%) showed a highly elevated level of serum ADA reaching 18.7 µg/mL. This finding aligns with existing literature supporting the association between higher ADA levels and the attainment of biologic remission (Kutlu et al. 2021). This patient had experienced side effects such as hair loss, and skin lesions, which led to the physician’s decision of drug discontinuation.

In recent systematic reviews and meta-analyses, the effectiveness of IFX and ADA for patients with CD and UC was examined. The findings indicated that both medications proved to be successful in treating patients with CD and UC (Lee et al. 2021; Liefferinckx et al. 2020) which is consistent with our findings in Egyptian IBD patients.

The finding of this study reveals a significant reduction in disease activity, as evidenced by lowered levels of CRP in both IFX and ADA-treated patients and this was in agreement with three recent studies (Kutlu et al. 2021; Maaser et al. 2019; Mizoshita et al. 2017).

The effectiveness of both IFX and ADA in CD patients was assessed by the significant decrease in the CDAI’s scores after treatment when compared with baseline values. This reduction aligns with the existing literature, as documented by Paweł Kawalec et al. (Mogilevski and Sparrow 2018).

Yong Il Lee et al. 2021 reported a significantly lower DAI score after treatment when compared with baseline values in patients receiving either IFX or ADA (Nielsen et al. 2022) and this coincides with our data.

In our study, there was a significant decrease in sTREM-1 levels following treatment with both IFX and ADA. This result was reported by Verstockt et al., 2019, confirming a shared consensus regarding the anti-inflammatory effects of these biologics (Okobi et al. 2021). On the contrary, Kutlu et al., 2021 reported a conflicting result with the present study on sTREM-1 levels (Pabla and Schwartz 2020) This discrepancy underscores the complexity of the inflammatory response and highlights the need for further investigation to unravel the nuanced effects of IFX and ADA on sTREM-1 in the context of IBD.

Our study also compared the efficacy of ADA and IFX in treating CD and UC. In UC patients,

both ADA and IFX demonstrated comparable efficacy. This result is consistent with the results of recent clinical trials (Papamichael et al. 2019; Patel and Yarur 2023; Plevris et al. 2019; Prins et al. 2021). On the contrary, a clinical trial and a meta-analysis had reported conflict results with our data (Lee et al. 2021; Razzaq 2017).

Our study found that ADA was more effective than IFX in treating CD patients. However, other studies have shown similar effectiveness between IFX and ADA in treating CD patients (Plevris et al. 2019; Seyedian et al. 2019; Shamkh et al. 2022). This difference in results may be due to the smaller size of our study group and the diverse ethnic backgrounds of the patients included in the previous studies.

In addition, our study involved 7 CD patients treated successfully with ADA, 4 of them had previously experienced treatment failure with IFX. This finding provides additional support for the superior efficacy of ADA over IFX. This result is consistent with previous research (Singh et al. 2021; Sostegni et al. 2003; Sturm et al. 2019; Su et al. 2019).

Our study has identified mild adverse events in patients receiving either IFX or ADA. In accordance with our data, other studies have shown a good safety profile for IFX and ADA in the management of moderate to severe IBD (Thorlund et al. 2015; Tursi et al. 2021; Verstockt et al. 2019).

The safety of biologic treatment during pregnancy is of crucial concern. Our unique observation of a successful pregnancy in an IFX-treated patient aligns with reassuring data from (Wang et al. 2022).

According to a recent study by Ole Haagen Nielsen et al. in 2022, it was discovered that biologics had more advantages than risks for pregnant women with IBD. The research also determined that the occurrence of negative pregnancy outcomes in IBD pregnant women using TNF inhibitors is not greater than that of the general population. (Yang et al. 2022).

## Limitations

One major limitation of our study is that we did not assess immunogenicity, specifically the presence of antibodies. In clinical practice, routine monitoring of serum trough levels and antibody formation at every visit is uncommon, primarily due to financial constraints. In additiony, the small sample size of our study is attributed to the low prevalence of IBD in Egypt.

## Conclusion

In conclusion, both IFX and ADA are safe and effective for treating moderate to severe IBD patients. It is important to monitor drug levels and manage potential side effects for best results. ADA is more effective than IFX in CD patients, while both treatments are equally effective in UC patients. These findings highlight the importance of individualization of IBD treatment to balance effectiveness and safety.

## Recommendation

To improve treatment strategies and overall quality of life for Egyptian patients with IBD, it is essential to conduct additional large-scale multicenter studies with longer follow-up periods. Baseline measurements of sTREM-1 are needed to accurately assess if treatment has an effect on this parameter.

## Data Availability

If you need data, just ask the corresponding author.
